# Cardiovascular Imaging Techniques in Systemic Rheumatic Diseases

**DOI:** 10.3389/fmed.2018.00026

**Published:** 2018-02-14

**Authors:** Fabiola Atzeni, Marco Corda, Luigi Gianturco, Maurizio Porcu, Piercarlo Sarzi-Puttini, Maurizio Turiel

**Affiliations:** ^1^Rheumatology Unit, University of Messina, Messina, Italy; ^2^Cardiology Unit, Brotzu Hospital, Cagliari, Italy; ^3^Cardiology Unit, IRCCS Galeazzi Orthopedic Institute, Milan, Italy; ^4^Rheumatology Unit, Luigi Sacco University Hospital, Milan, Italy

**Keywords:** systemic rheumatic diseases, coronary artery diseases, plasma asymmetric dimethylarginine, computed tomography, atherosclerosis, endothelial dysfunction

## Abstract

The risk of cardiovascular (CV) events and mortality is significantly higher in patients with systemic rheumatic diseases than in the general population. Although CV involvement in such patients is highly heterogeneous and may affect various structures of the heart, it can now be diagnosed earlier and promptly treated. Various types of assessments are employed for the evaluation of CV risk such as transthoracic or transesophageal echocardiography, magnetic resonance imaging (MRI), and computed tomography (CT) to investigate valve abnormalities, pericardial disease, and ventricular wall motion defects. The diameter of coronary arteries can be assessed using invasive quantitative coronarography or intravascular ultrasound, and coronary flow reserve can be assessed using non-invasive transesophageal or transthoracic ultrasonography (US), MRI, CT, or positron emission tomography (PET) after endothelium-dependent vasodilation. Finally, peripheral circulation can be measured invasively using strain-gauge plethysmography in an arm after the arterial infusion of an endothelium-dependent vasodilator or non-invasively by means of US or MRI measurements of flow-mediated vasodilation of the brachial artery. All of the above are reliable methods of investigating CV involvement, but more recently, introduced use of speckle tracking echocardiography and 3-dimensional US are diagnostically more accurate.

## Introduction

The risk of cardiovascular (CV) disease due to advanced atherosclerosis is higher in patients with systemic rheumatic diseases (SRDs) even in the absence of traditional CV risk factors (1), occurs earlier than in the general population, and is frequently asymptomatic in its early stages (2). The greater risk can be attributed to factors specifically related to autoimmune diseases such as chronic inflammation, disease duration and activity, and glucocorticoid, methotrexate, or antitumor necrosis factor-alpha (anti-TNFα) immunosuppression (3). The heart can be affected by various pathogenetic mechanisms involving the valves, coronary arteries, the conduction system, and the myocardium, endocardium, and pericardium, and their clinical manifestations include pericarditis, myocarditis and myocardial fibrosis, rhythm and conduction disturbances, coronaritis with ischemic heart disease, valve diseases, pulmonary hypertension, syncope, and diastolic or systolic heart failure (4).

Chronic inflammation is a major cause of atherosclerotic plaque (5) and endothelial dysfunction, which may be initially due to reduced nitric oxide (NO) bioavailability (6).

Non-invasive tests are the preferred means of investigating suspected or confirmed coronary artery disease (CAD), valve anomalies, and the other morphological and structural changes induced by SRDs (7, 8) (Table 1), but this review will also describe invasive methods of diagnosing CV involvement in SRD patients (Table 2).

**Table 1:** Imaging techniques for assessing cardiovascular involvement in patients with systemic rheumatic diseases.

**Measurements**	
1.Coronary arteries	
Invasive:	coronary diameter can be assessed using quantitative coronarography or intravascular ultrasound
Non-invasive:	coronary flow reserve can be assessed using transthoracic or transesophageal ultrasonography (US), magnetic resonance imaging (MRI), computed tomography, and positron emission tomography (PET) after endothelium-dependent vasodilatory provocation
2.Peripheral circulation	
Invasive:	strain-gauge plethysmography of an arm after intra-arterially infusing an endothelium-dependent vasodilator
Non-invasive:	flow-mediated vasodilation of the brachial artery measured by means of US or MRI

**Evaluation of general atherosclerotic alterations**	

1.Arterial stiffness parameters:	
Pulse wave analysis: augmentation index	
Pulse wave velocity	
2.Common carotid intima-media thickness (ccIMT) and carotid plaque analysis	
3.Determination of coronary calcium content	

**Table 2:** Imaging techniques and their correlations with pathological alterations in systemic rheumatic disease patients.

Imaging technique	Inflammation	Ischemia	Scarring	Vasculitis	Coronary arteries
Echo	No	Yes ± no	Yes ± no	Yes ± no	No
Nuclear	No	Yes ± no	Yes ± no	Yes ± no	No
Computed tomography	No	No	Yes ± no	No	Yes
CMRI	Yes	Yes	Yes	Yes	Yes ± no

## Sources and Selection Criteria

English-language articles published between January 1995 and July 2017 were found in the PubMed, Medline, and Cochrane Library databases using the key words “cardiovascular diseases” or “atherosclerosis,” “endothelial dysfunction,” “connective tissue disease,” “rheumatologic(al) disease” (including “rheumatoid arthritis,” “spondyloarthritis,” “systemic lupus erythematosus,” “Sjögren’s syndrome,” “mixed connective tissue disease,” “vasculitis”), “cardiovascular tools,” “non-invasive methods” or “invasive methods, “echocardiography,” and “coronary flow reserve.” The articles and book chapters in the papers’ reference lists were also reviewed.

## Methods of Evaluating Valve Abnormalities, Pericardial Diseases, and Ventricular Wall Motion Defects

### Transthoracic Echocardiography

Transthoracic echocardiography is a non-radiating, widely available, inexpensive, reliable, and easily reproducible means of precisely and non-invasively assessing pericardial diseases, valve anomalies, and defective ventricular wall motion, and Doppler analysis can be used to investigate valve function, pulmonary pressure, and diastolic left ventricular filling. Although it is operator dependent, limited in patients with a poor acoustic window, and does not allow the tissue characterization needed to detect inflammation, it is particularly valuable in diagnosing patients at high risk of developing CV disease such as those with rheumatoid arthritis (RA).

It was used by Rexhepaj et al. (9) to show that RA patients had significantly different early diastolic flow velocity (E), atrial flow velocity (A), and E/A ratios from those of control subjects, a finding indicating that, even when the size and thickness of their left ventricle and their myocardial performance are still normal, RA patients may have subclinically impaired left and right ventricular (LV and RV) function. Some authors (but not others) have found correlations between impaired diastolic function and disease duration and extra-articular manifestations, thus strengthening the hypothesis that chronic systemic inflammation is related to cardiac remodeling (10). This view is also supported by the fact that treatment with antitumor necrosis factor (TNF) agents improves diastolic function and normalizes left ventricle morphology within 6–12 months (11, 12).

Transthoracic echocardiography studies have shown that valve involvement is particularly frequent in patients with primary antiphospholipid syndrome (APS). There is a 32–38% prevalence of valve lesions, which mainly affect left-sided valves, valvular rings, the cordae tendineae, and other parts of the ventricular or atrial endocardium, and are characterized by diffuse leaflet thickening, valve stenosis, and often valvular regurgitation (13).

**Figure 1:**
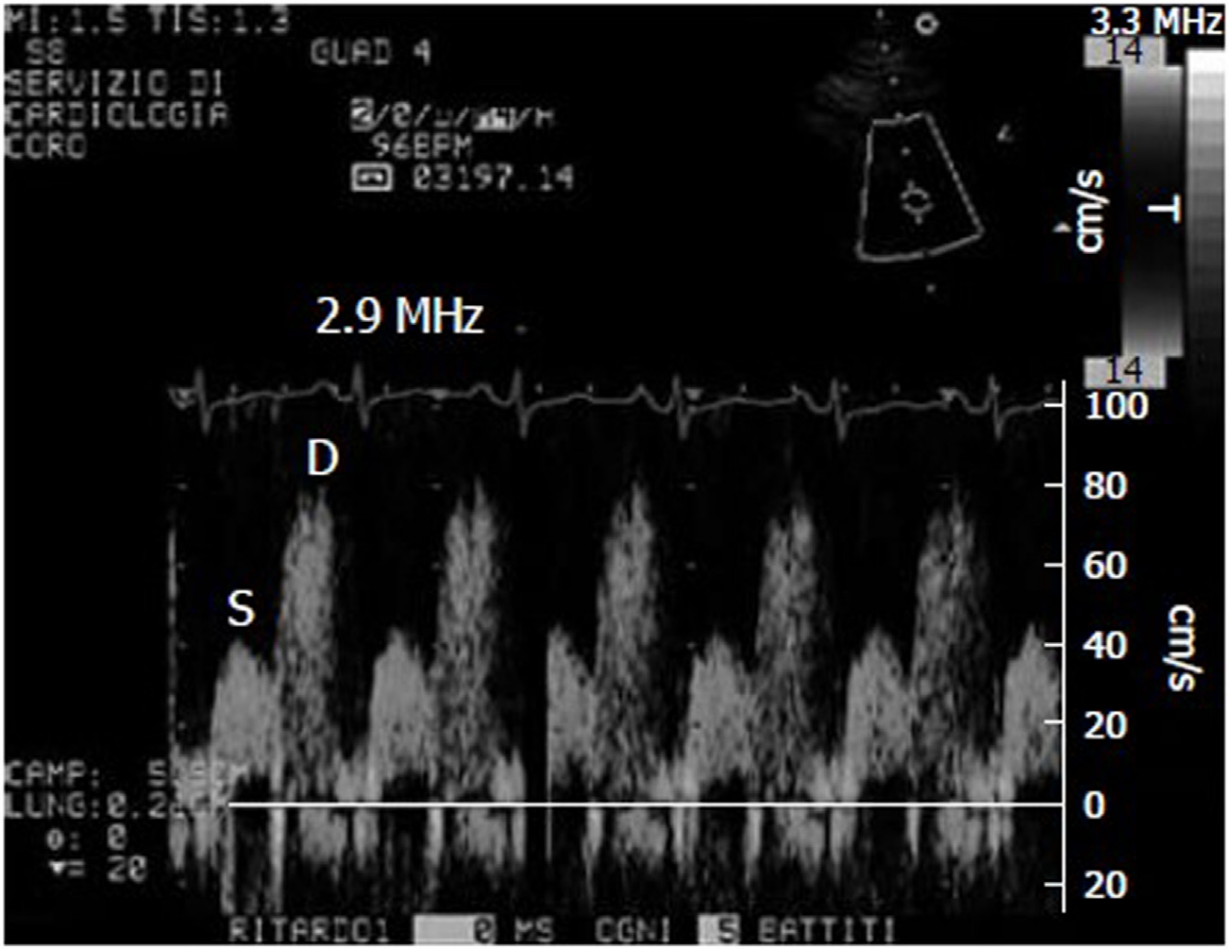
Example of coronary flow Doppler signal during dypiridamole-induced hyperemia. S, systolic flow; D, diastolic flow.

Doppler-derived mitral and pulmonary venous flow velocities used to be the only means of measuring diastolic LV filling, but the most widely used parameter for assessing diastolic function is now E/E’, which combines the findings of trans-mitral pulsed wave (PW) Doppler analysis with those of tissue Doppler imaging (TDI) (Figure 1).

### Transesophageal Echocardiography (TEE)

Transesophageal echocardiography is widely acknowledged to be more sensitive than TTE in detecting valve lesions and intracardiac masses (14), and Turiel et al. (15) used it to find that the prevalence of valve thickening, vegetation, and other sources of embolism was 61% in 56 patients with primary APS who were followed up for 5 years. Three-dimensional (3D) TEE has recently improved the diagnostic sensitivity of traditional 2D imaging by making it possible to view cross-sections of the mitral, aortic, and tricuspid valves (16). It also allows more reproducible and accurate measurements of LV volume, mass, and ejection fraction (LVEF), identifies wall motion abnormalities more precisely, and makes it possible to study the right ventricle, thus improving our understanding of valvular and sub-valvular abnormalities (17).

Turiel et al. (15) found valve thickening and/or regurgitation, vegetations or masses, and potential embolic sources in 33 out of 40 APS patients (82%), with the most frequent abnormality (63%) being mitral valve thickening. The number of associated and/or recurrent thromboembolic events was highest in the patients with anticardiolipin antibody titres of >40 GPL units, who also showed more TEE-revealed potential embolic sources than the patients with titres of <40 GPL units (*p* < 0.01). TEE can, therefore, be recommended in APS patients with clinical findings or high anticardiolipin antibody titres in order to characterize their cardiac abnormalities and detect possible sources of embolism (18), as well as to select the most appropriate treatment.

A recent TEE study found echo-generating mitral or aortic valve nodules, aortic atheromas, and mitral or aortic regurgitation in 30 unselected RA patients (19).

### Tissue Doppler Imaging

Tissue Doppler imaging, which has often been considered a reliable means of assessing myocardial deformations, can also be used to measure myocardial velocities, although its angle dependency means that they can only be used to detect deformities along the ultrasound beam, whereas the myocardium is simultaneously deformed in three dimensions (20).

The low doppler shift frequencies of the energy created by ventricular wall motion, which are filtered out in standard Doppler blood flow studies, can be recorded by means of pulsed Doppler imaging, whose temporal resolution makes it possible to analyze the temporal relationships between waves of systolic and diastolic myocardium velocity. Two-dimensional color doppler imaging has poor temporal resolution but good spatial resolution, which allows the differentiation of sub-endocardial and sub-epicardial velocity profiles, whereas the high temporospatial resolution of M-mode color-coded Doppler imaging only samples a single line. However, both require specific modifications to current ultrasound machines.

Tissue Doppler imaging is useful for studying CV involvement in patients with SRDs. Birdane et al. (21) found that, depending on their age and use of steroids, the diastolic biventricular function of RA patients is significantly impaired in comparison with healthy controls, and D’Alto et al. (22) found that SSc patients have both systolic and diastolic biventricular dysfunction, and that their systolic pulmonary artery systolic pressure (sPAP) is increased and further deteriorates after 3 years. Furthermore, Kama et al. (23) showed that patients with SSc have abnormal diastolic RV and LV function, and abnormal systolic RV function, with RV wall thickness being found to be the best individual predictor of global myocardial performance.

### Speckle Tracking Echocardiography (STE)

**Figure 2:**
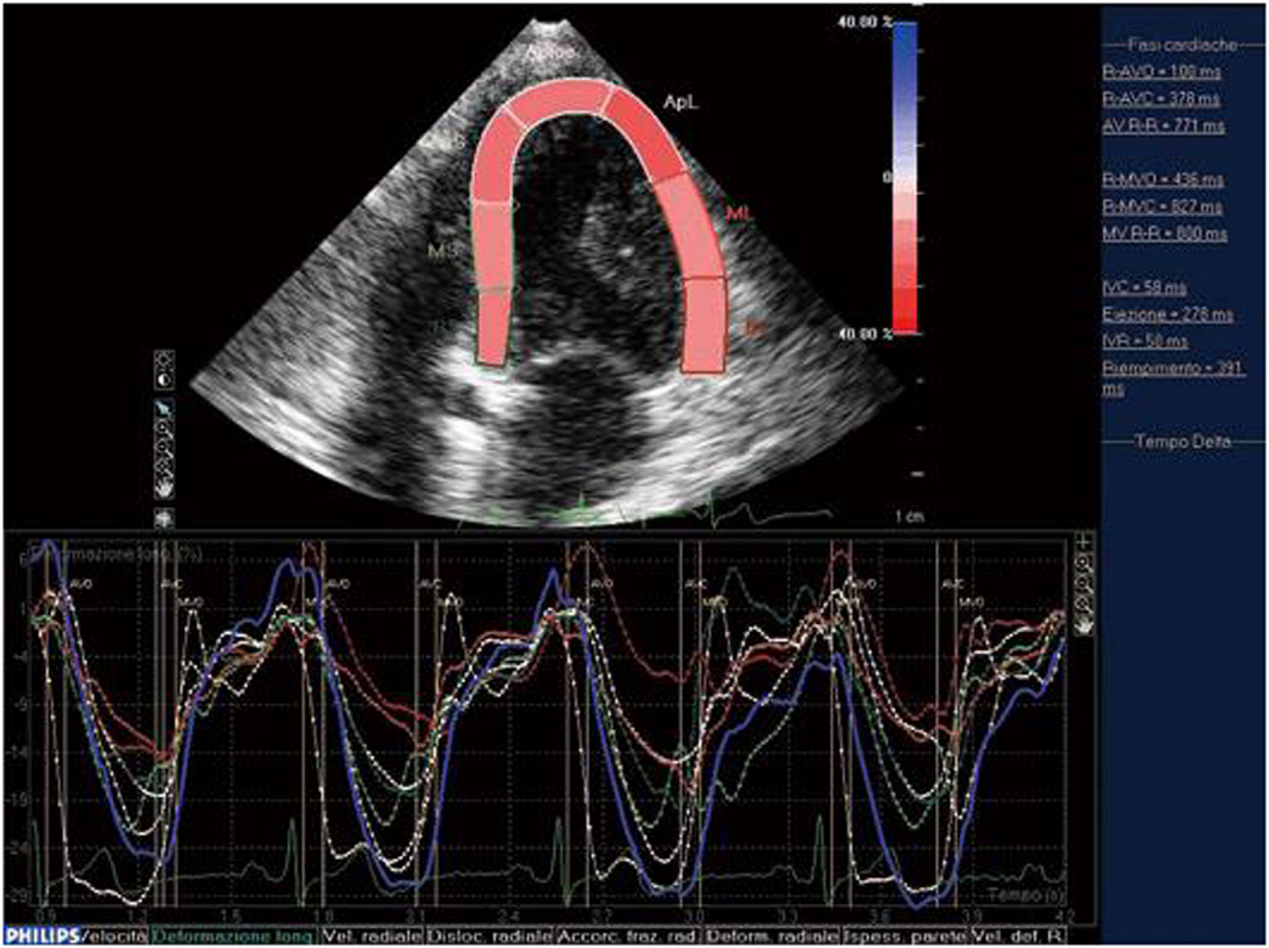
STE: systolic myocardial deformation after electromechanical activation. LV: longitudinal strain from the apical four-chamber view: time–strain curves show a negative end-systolic strain representing myocardial shortening during systole.

Speckle tracking echocardiography was introduced as a means of overcoming the limitations of TDI and allowing the evaluation of longitudinal, circumferential, and radial myocardial strain (9). It makes it possible to investigate regional myocardial deformation using the dimensionless parameter of strain (ε): i.e., the percentage change from the original size (24, 25). Furthermore, as myocardial motion is reflected by displacement, myocardial segments change position (displacement) but not their shape (deformation) if all of their parts have the same motion over a given period of time, but become deformed if different parts move differently. The use of both ε and displacement makes it possible to distinguish the passive movement and active contraction of each myocardial segment (26). As first described by Heimdal et al. (27), tissue deformation occurs during the cardiac cycle, and its rate (i.e., the strain rate, SR) is equivalent to the velocity gradient. Both TDI and STE can determine myocardial ε, but only angle-independent STE can accurately assess segmental myocardial deformation, which it does by means of gray scale-based, frame-by-frame image analysis. Furthermore, as myocardial ε can be tracked in two dimensions along the wall and not only along the ultrasound beam (22), it can be analyzed three dimensionally. When it is activated electro-mechanically, systolic myocardial deformation is reflected by radial thickening and longitudinal and circumferential shortening, thus making STE reliable in detecting slight cardiac involvement early in patients with connective tissue diseases (Figure 2) (24).

We have used STE strain analysis to find subclinical LV and RV abnormalities in RA patients (28). Yiu et al. (29) have shown that STE-revealed LV dysfunction is related to rhythm disturbances and the poorer functional capacity of patients with SSc, and Spethmann et al. (30) found that the systolic LV impairment of SSc patients with a preserved LVEF. is primarily due to alterations in the basal LV segments Finally, Atzeni et al. (31) have shown that STE-measured LV myocardial longitudinal ε is impaired in patients with primary Sjögren’s syndrome (pSS) in the absence of any clinical evidence of CV disease, and when traditional echocardiographic parameters were still negative, which suggests a myocardial alteration.

### Myocardial Contrast Echocardiography (MCE)

Myocardial contrast echocardiography is a new portable technique that uses microbubble contrast agents to visualize the coronary microvasculature and is ideal for non-invasively evaluating acute coronary syndromes (ACS) as it simultaneously assesses of regional wall motion and myocardial perfusion (32). Recent improvements in ultrasonography (US) technology and contrast agents means that MCE can now be used to assess chest pain, diagnose acute myocardial infarction, and establish its prognosis, assess successful reperfusion, and distinguish myocardial stunning and myocardial necrosis (32). Possible future applications include the detection of inflammation and ultrasound-induced thrombolysis in patients with ACS, but further studies are required in patients with SRDs.

### Cardiac MRI (CMRI)

**Figure 3:**
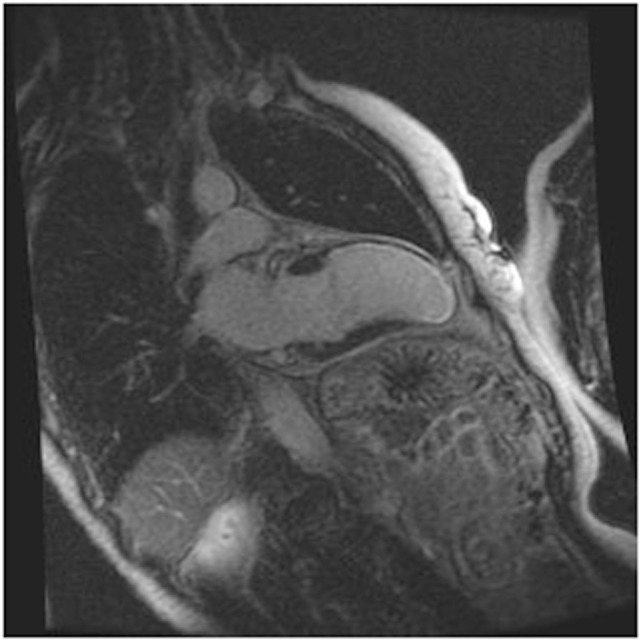
CRMI: a patient with previous anterior myocardial infarction. Late enhancement imaging showed transmural infarction in the left anterior descending artery.

Cardiac MRI non-invasively, accurately, and reproducibly assesses myocardial anatomy and function, and is now gold standard means of measuring LVEF, and LV and RV volumes (Figure 3) (8, 33–35). The images are constructed on the basis of signals produced by the many protons (hydrogen nuclei) that are mainly present in the body in the form of water molecules. The relaxation of the net proton vector can be attributed to two distinct processes that take place at the same time and can provide important information about human tissues: longitudinal (T1) and the transverse (T2) relaxation times. The basic pulse sequences used in CMRI are gradient-echo (GE) sequences that can form a cine loop and allow the assessment of wall motion and function; T1-weighted spin-echo sequences that are useful for anatomical imaging; T2-weighted spin-echo sequences that offer information about tissue edema. Late gadolinium-enhanced (LGE) images taken 15 min after the administration of the contrast agent allow the detection of myocardial fibrotic tissue (scarring), which appears as a bright area on a background of black myocardium (8, 34); non-invasive vessel angiography is also possible using a bolus injection of gadolinium.

Cardiac MRI can detect myocardial lesions in patients with mixed connective tissue disease (CTD) or cardiac symptoms such as myocardial infarction, inflammation, diffuse sub-endocardial fibrosis, and defective perfusion (34). It can also diagnose congenital heart disease and interstitial fibrosis in patients with dilated and infiltrative cardiomyopathies, assess the viability of the myocardium in patients with ischemic heart disease, reveal ventricular thrombi and constrictive pericarditis, measure valve dysfunction and iron overload in T2-weighted images, and assist in the evaluation of arrhythmogenic RV dysplasia cardiomyopathy (ARVD/C).

It is the gold standard means of evaluating myocardial viability. It has been clearly shown that LV dysfunction in patients with CAD may be related to myocardial stunning or hibernation, and can, therefore, be reversed (34). CMRI was used to compare myocardial structure and function in men and women with RA enrolled in the Evaluation of Subclinical Cardiovascular Disease and Predictors of Events in Rheumatoid Arthritis study and non-RA control subjects enrolled in the Baltimore Multi-Ethnic Study of Atherosclerosis, and showed that mean LV mass was 26 g (18%) less in the RA patients (*p* < 0.001) (36). There was no between-group difference in mean LV end-systolic and end-diastolic volumes, but mean LVEF, cardiac output, and stroke volume were also slightly lower in the RA group. These findings support the view that the progression to heart failure in RA patients may be due to reduced myocardial mass rather than hypertrophy.

LGE CMRI differentiates viable and non-viable myocardium and can identify which segments may need to be revascularised. It has also recently been shown that CMRI can identify vasculitis, myocarditis, and myocardial infarction in patients with symptomatic CTD and normal echocardiography findings (34).

CMRI-detected myocardial abnormalities are often encountered in RA patients with no known cardiac disease and, as they are associated with greater RA disease activity, inflammation may play a role in their pathogenesis (35). Furthermore, a T2-weighted and LGE CMRI study of global and regional morphology and systolic function in 94 RA patients with clinically diagnosed myocarditis showed greatly increased end-diastolic volumes, a reduced EF, thickened antero- and infero-lateral walls associated with reduced radial and longitudinal thickening, and a considerably higher T2 edema ratio and global LGE scores (37), thus suggesting that sustained inflammation gives rise to myocardial plasticity and deformation.

## Methods of Evaluating Coronary Arteries

### Stress Echocardiography

A pharmacological stress test is indicated for patients who cannot exercise adequately because of rheumatological, orthopedic, vascular, or pulmonary conditions. The various ways of evaluating coronary flow reserve (CFR) in such patients include transthoracic stress echocardiography with dipyridamole, which is cheap, simple, and non-invasive (Figure 1) (21, 38). CFR, which is assessed in the distal left anterior descending coronary artery, is the ratio between peak diastolic velocity during stress and at baseline. It is a highly sensitive (>90%) diagnostic marker of CAD (39) and highly specific when associated with regional wall motion analysis (40). A CFR of <2 indicates the presence of coronary stenosis (39), an abnormal reserve in the absence of a epicardial coronary stenosis, may indicate an impaired coronary microcirculation in patients with a reperfused myocardial infarction, hypertrophic cardiomyopathy, arterial hypertension with or without LV hypertrophy, diabetes mellitus, hypercholesterolemia, syndrome X, or other diseases (38). A reduced CFR correlates with a negative prognosis, and the range of CFR values is an independent prognostic indicator in patients with known or suspected CAD (41).

Hirata et al. (42) found that CFR was significantly lower in pre-menopausal women with systemic lupus erthymetosus (SLE) than in age- and gender-matched controls, and suggested that the microvascular impairment may be due to a functional endothelial alteration decreasing vasodilation as a result of pharmacological stress. Turiel et al. (43) found significantly lower CFR values in 25 untreated patients with early RA (ERA, a disease duration of <1 year) which, in the absence of wall motion abnormalities at rest and during pharmacological stress indicated the involvement of the coronary microcirculation and an association with endothelial dysfunction. CFR cannot distinguish micro- and macro-vascular CAD, but has additional diagnostic value over conventional wall motion analysis.

Ciftci et al. (44) have shown that RA patients have increased intima-media thickness (IMT) and reduced CFR. Chung et al. (45) found that the prevalence of more severe coronary calcification was higher patients with long-standing RA than in those with ERA, and Del Rincon et al. (46) demonstrated that ATS is more likely than in healthy controls. Accurate coronary microcirculation screening is therefore crucial in the earliest stages of RA, even if there are no signs or symptoms of CV involvement.

Endothelial dysfunction and increased carotid artery IMT are more prevalent in patients with psoriatic arthritis (PsA) but without any conventional CV risk factors or clinically evident CV disease than in controls (47). Our comparison of sub-clinical cardiac involvement in 22 outpatients meeting the Classification of Psoriatic Arthritis (CASPAR) Study Group criteria for PsA but no history of CV disease and 35 controls showed that the patients had higher asymmetric dimethylarginine levels and a significantly reduced CFR (48), and the significant correlation between these factors may indicate endothelial dysfunction and an impaired coronary microcirculation, thus confirming the view of Wakkee and de Jong (47) that active PsA is a risk factor for CV disease.

A Danish population cohort study by Ahlehoff et al. (49), which included 34,371 subjects with mild psoriasis, 2,621 with severe disease, and 607 with PsA. CV events were more frequent in the psoriasis patients, and their frequency increased with disease severity, and decreased with age at the time of disease onset. The risk was similar in the subjects with severe psoriasis alone and those with PsA. Given the high CV burden mainly related to endothelial dysfunction and ATS, an early diagnosis is fundamental in patients with PsA (47).

Our dypiridamole echocardiography study of 20 patients meeting the American College of Rheumatology (ACR) criteria for SSc but with no sign or history of CAD and 20 healthy volunteers showed that the former had a significantly lower CFR, which seems to support the idea that subjects with diffuse SSc have subclinical CV involvement (50). The findings were confirmed by Vacca et al. (51) who found that 19 out of 41 patients with SSc and no symptoms of CAD who underwent Doppler TTE with an adenosine infusion and dobutamine stress echocardiography had a reduced CFR, and 16 had wall motion anomalies.

All of the above findings underline the importance of detecting subclinical coronary microcirculation abnormalities, and CFR can be a useful marker in SRD patients. It seems that echocardiographic variables such as CFR can be reliable, sensitive, and specific means of detecting preclinical cardiac involvement relatively inexpensively.

### Computed Tomography (CT)

**Figure 4:**
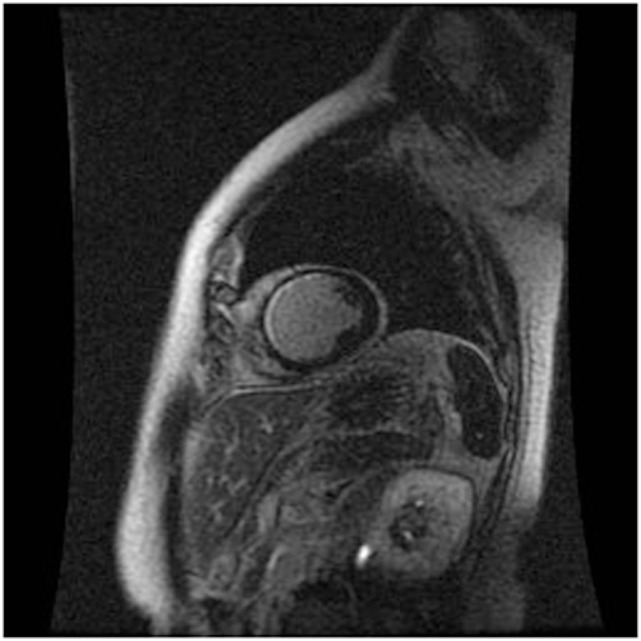
Cardiac CT: patient with high-grade left anterior descending artery stenosis (arrow).

Computed tomography has become a potent means of diagnosis that is almost indispensable in clinical practice (Figure 4). It is widely available, easy to use, non-invasive, and highly sensitive in detecting CAD and evaluating pericardial disease, pericardial fluid, and pericardial neoplasms. Given the small diameter lumens of coronary arteries, high resolution is critical, but the spatial and temporal resolution and volume coverage of modern multi-detector row CT systems are sufficient to allow robust imaging in many patients (Figure 4) (52).

When CT is used for the purpose of imaging coronary arteries, it is necessary to avoid unnecessarily high radiation doses (53). This issue has been addressed by recent advances in scanner technology and image sequencing, as well as randomized and controlled trials whose findings indicate that coronary CT angiography (CTA) has now become established as a means of assessing CAD (54). It can be carried out after the intravenous injection of an iodinated contrast agent (coronary CTA), or by simply using the contrast-free Agatston or coronary artery calcium (CAC) score (55), which correlates closely with total coronary calcium levels in histological samples, and measures early atherosclerosis. This score is recommended by the American College of Cardiology (56) in subjects with unclear results based on traditional algorithms of clinical CVD risk, and similarly by the European Society of Cardiology (ESC) (57).

**Figure 5:**
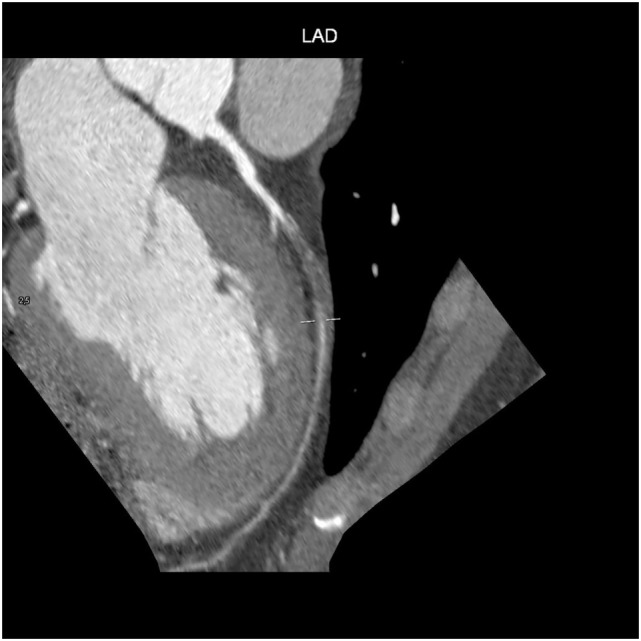
Coronary CT: chronic total occlusion of left anterior descending artery.

**Figure 6:**
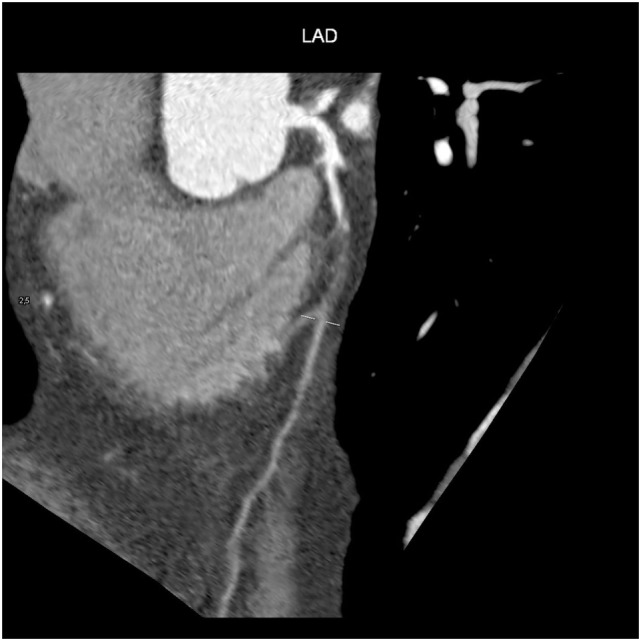
Coronary TC: chronic total occlusion of left anterior descending artery.

After the intravenous injection of a contrast agent, ≥64-slice CT can visualize the coronary artery lumen providing that the patients are carefully selected and prepared. Coronary CTA should only be considered for non-obese patients with a favorable calcium score who are in sinus rhythm, have a heart rate of ≤65 bpm (preferably ≤60 bpm), and can hold their breath sufficiently (58): short-acting β-blockers or other heart rate-lowering medications are recommended when necessary. However, as the specificity of coronary CTA decreases as coronary calcium increases (59), and given the high prevalence of coronary artery stenosis in symptomatic subjects with a score of >400 (60), coronary CTA is not recommended in patients with these characteristics (Figures 5 and 6) (61).

The diagnostic performance of coronary CTA is best in subjects with a low-intermediate pretest probability of having the disease, and it may, therefore, be useful to rule out coronary stenoses (62). It could also be used to assess CV risk in patients with RA as the current radiation exposure associated with CTA is ≤3–4 mSv, and the typical radiation dose associated with CAC scoring is <1 mSv. In patients with these characteristics, good quality images can be expected with reasonably little radiation exposure, but coronary CTA cannot exclude functional CAD.

Furthermore, newer developments such as fractional flow reserve (CT-FFR) require further validation.

Pericardial disease is frequently encountered alone or in association with other systemic disorders. Recognizing the typical manifestations of pericardial disease can be relatively easy, as these include acute pericarditis and an audible friction rub, and a reported retrosternal pain exacerbated by inspiration or being in the supine position. However, as pericardial disease can also be associated with non-specific symptoms and equivocal physical findings, it may be more difficult to diagnose without using CT, which is the most accurate means of imaging the calcified tissue associated with chronic pericarditis (63).

### Positron Emission Tomography (PET)

Positron emission tomography myocardial perfusion imaging, which detects the uptake of positron-emitting radiotracers in the heart, allows LV volumes to be precisely measured and used to determine myocardial perfusion (64, 65) on the basis of the LV as a whole or each of its standard segments. Decreased myocardial perfusion may indicate obstructive CAD or a reduced coronary microcirculation. PET–CT can identify high-risk ruptured atherosclerotic plaques in patients with symptomatic coronary and carotid artery disease, and PET can be used to predict CV mortality in patients with suspected angina or CAD (65). However, its use is limited by its cost, the availability of tracers, its use of ionizing radiation, and its restricted ability to assess cardiac structures.

## Non-Invasive Assessments of Arterial Involvement

### Carotid Ultrasonography

The ultrastructure of elastic arteries such as the carotid arteries can be easily investigated as they are close to the skin. A 7 MHz linear array transducer for B-mode US can be used to assess wall abnormalities, and the IMT of the far wall of the common carotid arteries [common carotid intima-media thickness (ccIMT)] is a major indicator of vascular aging (66) as an IMT of >1 mm is a reliable marker of generalized rather than localized carotid atherosclerosis (67). Increased ccIMT suggests a higher risk of myocardial infarction, peripheral arterial disease and stroke, but is only moderately sensitive in predicting future CV events (68). The measurement site is crucial for standardization and reliability but, although ccIMT assessments are considered to be quantitative, they do not allow detailed ultrastructural analysis of the whole extracranial carotid system (69): future myocardial and cerebrovascular events can be better predicted on the basis of the presence of plaques in the carotid system or total plaque area than on the basis of ccIMT, but their combination is recommended in asymptomatic adults at intermediate risk of CV events (70).

Many cross-sectional studies have found that patients with RA or AS have increased ccIMT indicating accelerated atherosclerosis, and patients with PsA, SLE, and SSc have been found to have subclinical atherosclerosis (71, 72). Furthermore, the findings of one small follow-up cohort study support the view that ccIMT can predict future CV events in patients with RA in the long term (73). Corrales et al. (74) divided 370 consecutive patients with established RA and no history of CV disease into groups considered to be at low, intermediate, high, or very high CVD risk on the basis of a modified risk score. All of them underwent carotid US but only 12% of the low-risk patients showed increased cIMT and/or carotid plaques indicating a high CV risk; however, these features were found in 65% of the patients at intermediate risk, and 85% of those at high or very high risk. CV imaging is, therefore, useful when evaluating patients at intermediate CV risk (74).

The current European League against Rheumatism (EULAR) guidelines indicate RA as an important risk factor for atherosclerosis, and so ccIMT may be useful for screening RA patients (75).

### Flow-Mediated or Nitroglycerine-Mediated Vasodilatation

Various studies have used flow-mediated (endothelium-dependent) vasodilatation (FMD) or nitroglycerine-mediated (endothelium-independent) vasodilatation (NMD) to assess early endothelial dysfunction in RA patients (76–78), and high-resolution US measurements of brachial artery FMD and NMD have shown both impaired and normal FMD in patients with rheumatic diseases (62). Reactive hyperemia leads to a significantly lower percentage increase from baseline FMD in RA patients than in healthy subjects (5.3 vs 8.3%), but there is no difference in the increase in NMD after nitroglycerine administration (18.3 vs 17.5%); impaired FMD has also been recorded in young RA patients with low disease activity scores (79).

The early endothelial dysfunction indicated by FMD% may precede the atherosclerosis revealed by ccIMT, and more marked dysfunction may accelerate the atherosclerotic process in RA patients (8, 33).

### Arterial Stiffness

Arterial stiffness, a surrogate measure of an increased risk of CV disease, reflects generalized vascular aging and atherosclerosis. The standard method of assessing overt atherosclerosis is to measure ccIMT; the parameters of arterial stiffness and wave reflection are pulse wave velocity (PWV), and transcutaneous measurememts of the augmentation index (AIx) (8, 33). Alternative methods include measuring aortic distensibility and the brachial-ankle elasticity index (baEI). One relatively small cross-sectional study of 113 RA patients found that patients in remission had significantly lower aPWV and AIx values than those with active disease (80). Another recent study of 138 RA patients has demonstrated that increased aPWV (as well as carotid plaque and cIMT) is predictive of CV events over a mean follow-up period of 5.4 years, with a hazard ratio of 1.85 per unit (m/s) increase in aPWV (81). The predictive value of the baEI in assessing CV risk has been widely studied in the general population and, although there are no published longitudinal outcome studies of RA patients, the findings of a meta-analysis suggest that they have a lower baEI (82).

New diagnostic techniques are needed to predict early endothelial dysfunction and overt atherosclerosis (33, 83) and identify patients at high risk of CV morbidity and mortality easily and in a cost-effective manner. It is likely that the new 3D carotid and aortic approaches will be a step in this direction.

## Conclusion

The increased CV mortality and morbidity in patients with SRDs is mainly due to atherosclerotic disease. There are various invasive and non-invasive means that can reliably screen, diagnose, and monitor the follow-up of CV manifestations in these patients. There is reasonable evidence to support the use of carotid US but, although newer imaging techniques such as CMRI and CT may improve risk stratification, there is still a lack of concrete data.

## Research Agenda

Carotid IMT has been specifically validated in cohorts of RA patients but only provides a single, non-dynamic measurement; it has not yet been shown that serial measurements are helpful in assessing ongoing CV risk as the results of small-scale prospective studies are conflicting. Although there are extensive prognostic outcome data concerning other diseases associated with a high risk of CV disease, the prognostic value of the baEI has not yet been assessed in longitudinal studies of RA patients. These aspects need to be evaluated and developed in these cohort of the patients.

The currently available clinical evidence concerning the use of imaging techniques in SRD patients supports the use of echocardiography but, although the findings of CMR, PET, CT, and hybrid imaging studies are promising, there is still a need for additional data before their clinical use can be recommended.

New means of individualizing treatment could lead to a more realistically achievable form of “personalized medicine” that would allow early and effective diagnostics, reduce hospitalization, and decrease morbidity and/or mortality in SRD patients. Bearing this in mind, cost effectiveness should not only be considered in the short term but also in the long term.

## Author Contributions

FA conceived the idea and wrote the manuscript. MC and LG participated to write the first draft. MC, MT, and PSP critically reviewed the paper.

## Conflict of Interest Statement

The authors declare that the research was conducted in the absence of any commercial or financial relationships that could be construed as a potential conflict of interest.
